# MED32/442: Internet Lectures: A five years experience at the University of Vienna

**DOI:** 10.2196/jmir.1.suppl1.e69

**Published:** 1999-09-19

**Authors:** H Kritz, C Najemnik, H Sinzinger

**Affiliations:** ^1^Univ. of Nuklearmedizin AKH Wien, ViennaAustria

**Keywords:** Internet lectures, Medical Education, Atherosclerosis, Webcasting

## Abstract

**Introduction:**

Internet lectures are a very useful specialized tool to distribute information to interested people. Our experience started 1994 and we want to give a survey.

**Methods:**

We started with a special lecture on atherosclerosis and improved the method within the last years. We had a lot of problems with certification and identification procedures, but they are solved now. In the last years we added web casting tools.

**Results:**

At this time we have 80- 140 student per term, which are participants of a lectures. These are students attending a certificate course. Additionally we distribute our content over two homepages which we have installed to improve our work http://www.lipidforum.at, http://www.billrothhaus.at (10000 hits/month).

**Discussion:**

Internet will be the most important tool for future teaching. It is an interesting experience to teach students from all over the world and you learn a lot from these people by yourself.

